# Influence of Cashew Agro‐Industrial By‐Product (*Anacardium occidentale*) and Cashew Tree Gum on the Properties of Sponge Cakes: A Fat Replacement Approach

**DOI:** 10.1111/1750-3841.70936

**Published:** 2026-02-26

**Authors:** Maria Eduarda Nobre do Nascimento, João Bruno Guilherme Mendes, Lais Viana Vasconcelos, Jessica Maria Silva Sousa, Celli Rodrigues Muniz, Daniele Maria Alves Teixeira Sá, Francisca Joyce Elmiro Timbó de Andrade

**Affiliations:** ^1^ Universidade Estadual de Campinas Campinas Brazil; ^2^ Instituto Federal de Educação, Ciência e Tecnologia do Ceará Fortaleza Brazil; ^3^ Embrapa Agroindústria Tropical Fortaleza Brazil

## Abstract

The processing of cashew apple in Brazil, particularly in the Northeast region, generates tons of by‐products. This scenario highlights a significant waste of material with bioactive potential. In addition to these, the cashew tree also produces cashew gum, a natural exudate with important technological applications. This study aimed to evaluate the impact of partial and total fat replacement with cashew tree gum and agro‐industrial residue from the cashew apple on the physicochemical, physical, microscopic, and sensory analysis of sponge cakes. To this end, three formulations were developed: A control formulation (F0), a formulation with 50% fat replacement (F50), and a formulation with 100% fat replacement (F100). In the raw batter, F50 and F100 showed significant differences compared to F0 in specific gravity (F0: 0.91 ± 0.01; F50: 1.22 ± 0.00; F100: 0.93 ± 0.04), viscosity (F0: 35397 ± 0.00 mPas; F50: 18415 ± 0.00 mPas; F100: 10782 ± 0.00 mPas), total bubble area (F0: 140.35 ± 0.00%; F50: 126.32 ± 0.00%; F100: 105.26 ± 0.00%), and cell density (F0: 2.91 ± 0.00 cells/cm^2^; F50: 13.84 ± 0.00 cells/cm^2^; F100: 33.56 ± 0.00 cells/cm^2^;) (*p* < 0.05). For baked cakes, only the specific volume of F0 (1.11 ± 0.12 mL g^−^^1^) and F100 (0.63 ± 0.19 mL g^−^^1^) differed significantly (*p* < 0.05). Sensory analysis indicated comparable acceptance and purchase intention between F0 and F50 for all attributes except color (F0: 8.17 ± 0.93; F50: 7.42 ± 1.25). F100 differed significantly in all attributes, but scores remained within the “Like” region of the hedonic scale, which goes up to 7 points. Based on the results, the formulations demonstrate technological potential, representing a functional and innovative food application.

**Practical Applications:**

This paper highlights the use of cashew by‐product and cashew gum as fat substitutes in sponge cakes, without affecting texture or sensory acceptance. It offers an innovative option for the food market focused on healthier, lower‐fat products.

## 1 Introduction

The cashew tree (*Anacardium occidentale* L.) provides a variety of products used across different sectors, including food production, timber, gums, nuts, pulps, and resins. During the processing of the cashew pseudofruit, approximately 20% of its weight is discarded, corresponding to its pomace, which is rich in nutrients, dietary fiber, and bioactive compounds, particularly vitamin C (Silva et al. 2018; Reina et al. 2022).

Products such as fruit pulps, sweets, and juices are commonly the end destination in fruit‐processing agro‐industries. However, within this production chain, by‐products often emerge that are treated as waste, leading to the loss of potentially valuable inputs rich in bioactive compounds. The Northeast region of Brazil, the country's leading cashew‐producing area, accounts for approximately 99% of national cashew nut production, large quantities of by‐products are generated. These by‐products have the potential to add value to the production process by reducing costs in the development of new products and, consequently, contributing positively to food security in the region (IBGE 2024; Alves et al. 2019; Cury et al. 2017).

Gums and dietary fiber stand out for their nutritional potential, as these are plant‐based materials with prebiotic effects. They play a role in maintaining cholesterol and glycemic levels and possess functional properties that enable their incorporation into food formulations as fat replacers, enhancing viscosity and water retention capacity (Andrade et al. 2018; Surampudi et al. 2016; Giuntini et al. 2022; Tolve et al. 2024).

The exudate extracted from the cashew tree, which contains cashew gum (CG), has attracted scientific interest due to its natural abundance, low cost, and wide range of applications, including biomedical and pharmaceutical uses. CG is characterized by its hydrophilicity, biocompatibility, biodegradability, and structural flexibility and even in higher proportions, could result in low‐viscosity emulsions with surface‐active properties (Porto et al. 2015; Padilha et al. 2020; Osano et al. 2014).

The growing demand for innovative food products offering functional benefits and improved quality of life for consumers is evident. In this context, the bakery industry offers a wide variety of products with distinct characteristics, which can be reformulated to meet evolving market demands. Previous works have incorporated dried cashew apple fiber as a value‐added ingredient in bakery products, including its use as a partial replacement for wheat flour in cakes (Adegunwa et al. 2020). However, these studies rely on energy‐intensive processing steps, such as drying and milling, before the fiber can be incorporated.

Accordingly, the present study aimed to analyze the centesimal composition, physical, microscopic, and sensory characteristics of sponge‐type cakes in order to evaluate the impact of fully or partially replacement of fat with cashew tree gum and agro‐industrial residue from the cashew apple.

## 2 Materials and Methods

Purified CG was prepared based on the method described by Sousa et al. (2019). The crude exudate was dehydrated at 45°C, resuspended in distilled water (10% w/v), and subjected to ethanolic precipitatation (3:1 v/v). The purifield material was allowed to dry in a fume hood to remove residual solvent and subsequentily pulverized using a mortar and pestle (Torquato et al. 2004).

Cashew apples were sanitized using a fruit‐appropriate sanitizing solution at a concentration of 100 ppm. The nuts were then removed, and the apples were pulped using a juicer (NATIONALIZER—HB5858R). The by‐product resulting from this process was subsequently used in sponge cake production, as described in Table 1.

**TABLE 1:** Formulation of cakes prepared with different levels of fat replacement by cashew agro‐industrial by‐product and cashew gum.

Ingredients ^a^	F0(%)	F50(%)	F100(%)
Wheat flour	100	100	100
Sugar	100	100	100
Eggs	50	50	50
Salt	0.5	0.5	0.5
Milk powder	6	6	6
Baking powder	4	4	4
Water	55	55	55
Fat	43	21.5	0
Cashew fiber	—	21.5	43
Cashew gum	—	0.8	0.8

*Note*: F0, control formulation (100% fat + 0% dietary fiber + 0% cashew gum); F50, 50% fat replacement (50% fat + 50% dietary fiber + cashew gum); F100, 100% fat replacement (0% fat + 100% dietary fiber + cashew gum).

^a^ Flour‐based reference formulations.

### 2.1 Batter Specific Gravity

The specific gravity of the batters was measured using a container with the batter and was weighed. Specific gravity was calculated by dividing the weight of the batter by the weight of the same volume of water. All measurements were performed in triplicate at room temperature (28 ± 2°C), and used the method of Andrade et al. (2018).

### 2.2 Viscosity

Viscosity analysis was performed using a microprocessed rotational viscometer (QUIMIS‐Q860M21) at a temperature of 24 ± 2°C. Spindle 3 was used at a speed of 17 rpm for the control sample, while spindle 4 was used for the other formulations, operating at 17 rpm for sample F50 and 28 rpm for sample F100. All analyses were conducted in triplicate.

### 2.3 Microscopic Analysis of Air Bubbles in the Batter

The slides with the batter were observed in an optical microscope (OPTON‐TIM‐2005 B, Brazil) using a 10× objective lens. The software Mias (Electronic Eyepiece, 2008) was used to analyze air bubble distribution through the fraction of total bubble area (AF %) and cells/cm^2^ (CD).

The procedure was conducted according to the method of Andrade et al. (2018).

### 2.4 Proximate Composition

For the proximate composition were used the AOAC methods. The content of moisture was gravimetrically determined in an oven at 105°C until constant weight. Ash content was determined using a muffle furnace at 550°C. Lipid content was determined using the Soxhlet extraction method using hexane under heating for 8 h. After extraction, the flasks were placed in an oven at 105°C. The samples were then weighed, and lipid content was calculated as a percentage. Crude protein was calculated using 6.25 as the conversion factor from determined nitrogen. Carbohydrate content was obtained using the difference method.

### 2.5 Cake Specific Volume

In the analysis was measured using sesame seeds by the method of Andrade et al. (2018), calculated by the quotient of volume and mass at room temperature (28 ± 2°C). The tests were performed in triplicate and with results reported in mL g^−^^1^.

### 2.6 Scanning Electron Microscopy

Samples were fixed in Karnovsky solution diluted in 0.2 M cacodylate buffer (pH 7.2) for 48 h under refrigeration. Subsequently, they were washed three times with 0.2 M cacodylate buffer for 10 min each, post‐fixed in 1% osmium tetroxide for 1 h, washed in distilled water, and dehydrated through a graded ethanol series (20, 30, 40, 50, 60, 70, 80, and 90%) for 15 min each. Final dehydration was performed in 100% ethanol, three times for 15 min each. The samples were then dried in a critical point dryer (EMS 850), mounted on aluminum stubs, and sputter‐coated with platinum. Finally, they were examined using a TESCAN scanning electron microscope (model VEGA 3 SBU) at an accelerating voltage of 15 kV for image acquisition (Bozzola and Russell 1999).

### 2.7 Color

The color of the cake crumb was determined using the Commission Internationale de l'Éclairage (CIE) Lab* color system with a portable colorimeter (Delta Color, Colorium 2). Readings were taken in four replicates for each formulation at the grounded sample. The results were determined by the device according to the Hunter Lab color scale (L*a*b*). The data were processed using Lab7 software.

### 2.8 Sensory Analysis

The Sensory Analysis Laboratory of the Federal Institute of Ceara (IFCE), Sobral Campus, was used, following approval by the Research Ethics Committee involving human subjects (CEP—Plataforma Brasil) (CAAE No. 34094920.0.0000.5589, IRB Approval No. 4.142.175). A sensory acceptance test was performed in individual booths with 120 untrained panelists, both male and female, aged between 18 and 60 years, participants consisted of students and staff of IFCE. All participants were regular consumers of cakes. Cake samples were served according to a randomized block design. Each sample was served at approximately 28°C in odorless, 50 mL plastic cups coded with random three‐digit numbers. During the session, water and plain crackers were provided to cleanse the palate between samples. Samples were evaluated for acceptability based on the attributes of color, aroma, texture, flavor, and overall impression, using a nine‐point structured hedonic scale, with 1 representing “dislike extremely” and 9 representing “like extremely.” Purchase intent was also assessed by the same evaluators using a five‐point scale ranging from 5 (“would certainly buy”) to 1 (“would certainly not buy”).

### 2.9 Texture Profile Analysis

Crumbs from three cake batches were evaluated for texture (Texture Profile Analysis [TPA]) using TA‐XT Plus Texture Analyzer (Stable Micro Systems, UK). The methodology described by Andrade et al. (2018) and AACC 74‐09.01. Using a 2 kg load cell and a 36 mm probe, 18 samples (30 mm^3^ each, without crumbles) were subjected to dual compression (50% strain, 1 mm s^−1^ speed, 5 s delay). Firmness, springiness, cohesiveness, and chewiness were derived from the resulting force‐time curves. All measurements were taken at room temperature after one day of storage, and values are presented as mean ± standard deviation.

### 2.10 Statistical Analysis

A one‐way ANOVA followed by Tukey's test was used to evaluate differences between groups ($*p* < 0.05$). These statistical procedures were performed using Statistica software version 7.0, and results were considered significant at a 95% confidence level.

## 3 Results and Discussion

Fat plays multiple roles in a sponge cake, including entrapping air bubbles, contributing to softness, flavor, among others, being a highly caloric ingredient. To evaluate the batter of the formulations listed in Table 1, the analyses described in Table 2 were conducted.

**TABLE 2:** Effects of fat replacement by cashew agro‐industrial by‐product and cashew gum on the specific gravity and air bubble distribution in the batters.

Sample	Specific gravity	Viscosity (mPas)	CD (células/cm^2^)	AF (%)
F0	0.91 ± 0.01^c^	35397 ± 0.00^a^	140.35 ± 0.00^a^	2.91 ± 0.00^c^
F50	1.22 ± 0.00^a^	18415 ± 0.00^b^	126.32 ± 0.00^b^	13.84 ± 0.00^b^
F100	0.93 ± 0.04^b^	10782 ± 0.00^c^	105.26 ± 0.00^c^	33.56 ± 0.00^a^

*Note*: Values are expressed as mean ± SD. Equal letters within the same column indicate no statistically significant difference according to Tukey's test at 5% significance level. F0, control (100% fat + 0% fiber + 0% cashew gum); F50, 50% fat replacement (50% fat + 50% fiber + cashew gum); F100, 100% fat replacement (0% fat + 100% fiber + cashew gum); CD, number of bubbles per cm^2^ (cell density, cells/cm^2^); AF, percentage of total bubble area/total image area.

Specific gravity is a measure that evaluates the air‐holding capacity of the cake batter over the course of mixing and gas release by the leavening agent. Enhanced air incorporation leads to lower specific gravity (Psimouli and Oreopoulou 2013). The sample F100 showed a statistically different specific gravity behavior compared to the other samples. High specific gravity values are undesirable in relation to cake volume, causing a dense crumb with a compact and inadequate appearance (Andrade et al., 2018). Lower specific gravity indicates higher air incorporation in the batter, resulting in cakes with greater softness (Psimouli and Oreopoulou 2013). However, as demonstrated in Table 7, texture analysis based on hardness revealed that softness was not affected despite the reduction in specific gravity values, since the hardness was statistically equal to the control sample (F0), which contained 100% fat. Thus, the softness typically imparted by fat, a desirable characteristic in cakes, was also observed in F100, highlighting that fat replacement was effective in maintaining the expected softness in a fat‐containing cake.

According to Sampaio et al. (2015), viscosity can be defined as the resistance a fluid presents to flow over a surface. A statistically significant difference was observed among the three samples, showing that as fat replacement increases, the viscosity decreases. Thus, changes in product composition reduced the viscosity of the samples, corroborating the findings of Carlos et al. (2019), who reported that agents such as fats are substances used to enhance flavor and creaminess in preparations, often increasing the viscosity of the product, therefore, their substitution may lead to a viscosity reduction. In addition, Botrel et al. (2017) and Andrade et al. (2013) emphasize that gums, such as CG, are natural polymers and important agents capable of modifying the viscosity of liquids, acting as non‐Newtonian fluids with pseudoplastic characteristics.

The partial or total replacement of fat by CG and fibers from the cashew peduncle resulted in a reduction in the number of bubbles per cm^2^ (CD) (Table 2), but the bubbles increased in size. Even though samples F50 and F100 contained fewer bubbles, they exhibited a higher percentage of bubble area (AF). During the preparation of sponge cake batter, bubbles are formed as a result of air incorporation into the batter. Both the applied method and the physicochemical attributes of the ingredients, such as surface tension and viscosity, play a critical role in determining the effectiveness of batter aeration (Andrade et al. 2018).

The control sample (Table 2) (F0) showed better distribution of air bubbles (CD), indicating that partial or total replacement of fat with CG and cashew peduncle fibers led to fewer bubbles (CD) as well as an increase in the air bubble area (AF) in samples F50 and F100. This can be confirmed by the specific volume (Table 3), where F0 had a specific volume equal to F50, and F50 was statistically similar to F100. The larger bubble area may have caused the difference in specific volume between F100 and F0, as the formulation with total fat replacement presented larger bubbles which, if ruptured during baking, could significantly affect the cake volume. A higher quantity of uniformly distributed, smaller air bubbles is desirable to ensure adequate cake texture and volume (Hicsasmaz et al. 2003; Jia et al. 2014).

**TABLE 3:** Effects of fat replacement by cashew agro‐industrial by‐product and cashew gum on the specific volume.

Sample	Specific volume (mL g‐^1^)
F0	1.11 ± 0.12^a^
F50	0.81 ± 0.23^ab^
F100	0.63 ± 0.19^b^

*Note*: Values are expressed as mean ± SD. Equal letters within the same column indicate no statistically significant difference according to Tukey's test at 5% significance level. F0, control (100% fat + 0% fiber + 0% cashew gum); F50, 50% fat replacement (50% fat + 50% fiber + cashew gum); F100, 100% fat replacement (0% fat + 100% fiber + cashew gum).

Authors Lin et al. (2017), Sahagún et al. (2018), and Giarola et al. (2021) identify viscosity as a critical factor in the formation of stable and efficient air bubbles. However, they also report that excessively high viscosity can result in a batter that is difficult to mix, reducing air incorporation. In this context, the statistical differences in batter viscosity, influenced by different aeration agents such as fat or gum, may have had a direct impact. The simultaneous use of both agents in the same batter, as in sample F50, resulted in an acceptable number of air bubbles, though distributed irregularly. This suggests that total fat replacement with CG may be the most effective strategy in this case, indicating that maybe the use of a single type of stabilizing agent allows for more efficient air retention during mixing.

Table 3 presents the data related to the specific volume of the batters, which results from the formation of air bubbles during batter preparation.

**FIGURE 1:**
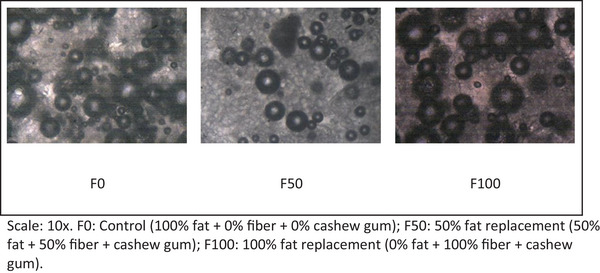
Effect of fat replacement by cashew agro‐industrial by‐product and cashew gum on the distribution of air bubbles in the batters. Scale: 10x. F0: Control (100% fat + 0% fiber + 0% cashew gum); F50: 50% fat replacement (50% fat + 50% fiber + cashew gum); F100: 100% fat replacement (0% fat + 100% fiber + cashew gum).

According to Psimouli and Oreopoulou (2012) and Barbosa (2019), specific volume indicates the degree of batter development associated with its porous structure. Thus, an excessively high or low volume may suggest flaws in the production process. It was observed that the control sample (F0) (Table 3) exhibited the highest specific volume, while sample F50 showed statistical similarity to both F0 and F100, which were statistically different from each other. Kırbaş et al. (2019) found that increasing the amount of fiber in cake formulations leads to a reduction in volume, resulting in differences compared to the control sample. This behavior, when compared with the analysis of air bubbles (Figure 1 and Table 2) and specific gravity (Table 3) in sample F100, showed that the bubbles were fewer and larger. This suggests that such bubbles may rupture due to the absence of fat, which typically helps stabilize them. Fat replacers may contribute to bubble formation but may not be as effective in stabilizing them. Consequently, sample F50 exhibited a higher specific volume than F100, likely due to the presence of 50% fat in its formulation.

A reduction in specific volume (Table 2) was observed to be directly proportional to the decrease in batter viscosity and inversely proportional to specific gravity (Table 3). In other words, the higher the viscosity, the greater the bubble formation and specific volume, and the lower the specific gravity. More detailed insights into the behavior of the microparticles are provided by scanning electron microscopy (SEM), as shown in 2. Andrade et al. (2018), when replacing fat in cakes with galactomannan, observed similar results in their experiments, lower viscosity led to reduced air incorporation during batter preparation.

**FIGURE 2:**
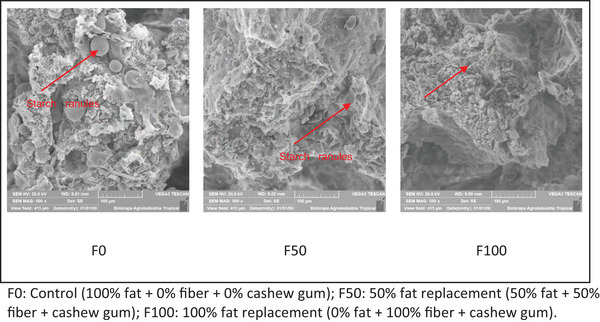
Effects of fat replacement by cashew agro‐industrial by‐product and cashew gum on the cake microstructure. F0: Control (100% fat + 0% fiber + 0% cashew gum); F50: 50% fat replacement (50% fat + 50% fiber + cashew gum); F100: 100% fat replacement (0% fat + 100% fiber + cashew gum).

Fat replacement reduced the specific volume of the cakes (Table 3). Accordingly, it was also observed that viscosity (Table 2) followed the same trend, being directly proportional to specific volume. It is also possible that the reduction in viscosity may be related to the decrease in starch granule size, as evidenced by SEM (Figure 2).

Table 4 presents the proximate composition of the cakes, which is essential for evaluating both the technological and nutritional characteristics of the samples.

**TABLE 4:** Effects of fat replacement by cashew agro‐industrial by‐product and cashew gum on the proximate composition and total energy value of the cakes.

	F0	F50	F100
Moisture	27.63 ± 0.15^c^	30.19 ± 0.06^b^	31.27 ± 0.47^a^
Ash	1.11 ± 0.01^a^	1.03 ± 0.01^b^	1.07 ± 0.02^ab^
Lipids	10.64 ± 0.17^a^	6.97 ± 0.13^b^	2.26 ± 0.25^c^
Proteins	4.45 ± 0.13^c^	5.62 ± 0.20^b^	6.12 ± 0.05^a^
Carbohydrates	56.18 ± 0.11^b^	56.96 ± 1.52^ab^	59.28 ± 0.53^a^

*Note*: Values are expressed as mean ± SD. Equal letters within the same column indicate no statistically significant difference according to Tukey's test at 5% significance level. F0, control (100% fat + 0% fiber + 0% cashew gum); F50, 50% fat replacement (50% fat + 50% fiber + cashew gum); F100, 100% fat replacement (0% fat + 100% fiber + cashew gum).

Fat replacement led to an increase in moisture content in formulations (Table 4) F50 and F100, while, as expected, reducing the lipid content. Samples F50 and F100 contain a higher proportion of moist by‐product in their composition, which could be responsible for the observed increment in moisture. Higher carbohydrates and protein content was also observed in samples F50 and F100. This may be attributed to the removal of fat which contains neither carbohydrates nor proteins, and the addition of a by‐product with both macronutrients, thereby contributing to their higher levels in the formulations.

The microscope enables the visualization of structures that cannot be detected by the human eye. SEM is a technique used to evaluate the morphology and identify the chemical elements of solid samples, and is recognized for its extensive applicability across diverse matrices for the observation and analysis of microstructural information in solid objects (Dedavid et al. 2007). As shown in Figure 2, larger starch granules (SG) were observed in the control formulation (F0), and a progressive reduction in SG size was noted in samples F50 and F100, in accordance with the degree of fat replacement. The lower the fat content, the smaller the SG observed, which could be related to the decrease of the viscosity (Table 2).

An important factor affecting cake acceptance is color. As shown in Table 5, the samples with fat replacement by cashew by‐product and CG presented similar lightness (L*) compared to the control sample (F0). However, regarding the chromaticity parameters *a* and *b*, sample F0 differed from the others, exhibiting a greater tendency towards reddish and yellowish tones.

**TABLE 5:** Effects of fat replacement by cashew agro‐industrial by‐product and cashew gum on the color of cakes.

	L*	a*	b*
F0	69.78 ± 1.87^a^	1.59 ± 0.25^b^	24.29 ± 1.02^a^
F50	63.97 ± 1.97^a^	5.88 ± 0.44^a^	20.53 ± 1.32^b^
F100	62.98 ± 3.34^a^	6.46 ± 0.72^a^	18.89 ± 2.36^b^

*Note*: Values are expressed as mean ± SD. Equal letters within the same column indicate no statistically significant difference according to Tukey's test at 5% significance level. F0, control (100% fat + 0% fiber + 0% cashew gum); F50, 50% fat replacement (50% fat + 50% fiber + cashew gum); F100, 100% fat replacement (0% fat + 100% fiber + cashew gum).

Sabino et al. (2017), who developed cookie‐type biscuits using cashew‐derived by‐products, observed that the application of dehydrated cashew by‐products in culinary preparations typically imparts darker coloration to the products, generally tending towards brown shades. Considering the reddish coloration of cashew residues, these differences in color compared to commercial products—which usually have lighter tones—were noted, without negatively affecting other sensory characteristics.

All attributes evaluated in the sensory acceptance test received scores between 7 and 8 (“liked it”) (Table 6), with sample F50 being statistically similar to the control sample for all attributes except color, in which the control sample showed higher acceptability. This finding may be related to the increased red and yellow hues in the cakes due to the addition of the cashew by‐product (Table 5). Some authors, such as Eggleston et al. (2018) and Stavale et al. (2019), report that carbohydrates can act as texture modifiers and aroma enhancers, which may explain the difference in acceptance between samples F0 and F50 compared to sample F100, which had total fat replacement and a higher amount of cashew by‐product.

**TABLE 6:** Effects of fat replacement by cashew agro‐industrial by‐product and cashew gum on the acceptance of cakes.

Attributes	F0	F50	F100
Overall impression	7.97 ± 1.07^a^	7.71 ± 1.05^a^	7.14 ± 1.13^b^
Aroma	7.55 ± 1.35^a^	7.61 ± 1.32^a^	7.08 ± 1.49^b^
Color	8.17 ± 0.93^a^	7.42 ± 1.25^b^	7.00 ± 1.37^c^
Flavor	7.77 ± 1.32^a^	7.84 ± 1.19^a^	7.23 ± 1.31^b^
Texture	8.02 ± 1.22^a^	7.84 ± 1.14^a^	7.30 ± 1.50^b^
Purchase intention	4.50 ± 0.70^a^	4.50 ± 0.80^a^	4.00 ± 0.97^b^

*Note*: Values are expressed as mean ± SD. Equal letters within the same column indicate no statistically significant difference according to Tukey's test at 5% significance level. F0, control (100% fat + 0% fiber + 0% cashew gum); F50, 50% fat replacement (50% fat + 50% fiber + cashew gum); F100, 100% fat replacement (0% fat + 100% fiber + cashew gum).

Sample F100, containing the highest percentage of cashew by‐product, showed statistically significant differences compared to the other samples, confirming the possible flavor differentiation due to the addition of by‐products. This finding corroborates Cordero‐Soto et al. (2024), who observed that besides enhancing nutritional value, the incorporation of by‐products in formulations can intensify flavors.

Regarding purchase intention (Table 6), all formulations were statistically similar, ranging between “Probably would buy” and “Definitely would buy,” emphasizing the feasibility of replacing fat with cashew by‐product and CG.

The replacement of fat by cashew agro‐industrial by‐products did not affect the firmness, elasticity, and chewiness parameters of the cakes (Table 7). According to Andrade et al. (2018) and Pathaw et al. (2021) firmness is a critical indicator of cake quality, it encompasses attributes such as softness and lightness and is quantitatively defined by the resistance of the matrix to compression. Elasticity measures the capacity of the sample to recover after compression. Most products typically lose elasticity over time, while chewiness refers to the number of chewing cycles required to swallow the food (Andrade et al. 2018; Pathaw et al. 2021; Movahhed et al. 2020). Cohesiveness was the only parameter that showed a statistically significant difference, with sample F0 being equal to F50 but different from F100, while F50 and F100 were statistically similar. Cohesiveness indicates the internal bonds strength within the internal matrix of the sample and is related to firmness as well as lower specific volume values (Pathaw et al. 2021; Fatah‐Jahromi et al. 2024). Thus, it can be inferred that the replacement of fat by cashew by‐product and CG did not significantly impact the cake texture.

**TABLE 7:** Effects of fat replacement by cashew agro‐industrial by‐product and cashew gum on the texture of cakes.

	F0	F50	F100
Hardness	0.03 ± 0.00^a^	0.03 ± 0.00^a^	0.03 ± 0.01^a^
Elasticity	0.82 ± 0.03^a^	0.82 ± 0.01^a^	0.82 ± 0.02^a^
Chewiness	0.02 ± 0.00^a^	0.01 ± 0.00^a^	0.02 ± 0.00^a^
Cohesiveness	0.56 ± 0.02^a^	0.59 ± 0.01^ab^	0.66 ± 0.04^b^

*Note*: Values are expressed as mean ± SD. Equal letters within the same column indicate no statistically significant difference according to Tukey's test at 5% significance level. F0, control (100% fat + 0% fiber + 0% cashew gum); F50, 50% fat replacement (50% fat + 50% fiber + cashew gum); F100, 100% fat replacement (0% fat + 100% fiber + cashew gum).

## 4 Conclusion

It is evident that both partial and total replacements of fat by cashew by‐product and CG exhibited hardness, elasticity, and chewiness comparable to those of the control. Furthermore, the results indicate an acceptable performance regarding sensory analysis acceptance, and purchase intention, while reducing viscosity. This study highlights the innovative potential compared to products currently available on the market, offering a lower caloric value due to fat reduction, which is also relevant for the product's healthfulness, meeting consumer demand for healthier options. This work presents an innovation in the food production sector, with great potential for market integration, valorizing cashew as a national product.

## Author Contributions

**Maria Eduarda Nobre do Nascimento**: conceptualization, investigation, writing – original draft, methodology, validation, visualization, writing – review and editing, formal analysis, data curation. **João Bruno Guilherme Mendes**: conceptualization, investigation, writing – original draft, methodology, formal analysis, data curation. **Lais Viana Vasconcelos**: conceptualization, investigation, writing – original draft, formal analysis. **Jessica Maria Silva Sousa**: conceptualization, investigation, methodology, formal analysis. **Celli Rodrigues Muniz**: methodology, data curation, formal analysis. **Daniele Maria Alves Teixeira Sá**: conceptualization, investigation, writing – original draft, methodology, validation, visualization, writing – review and editing, project administration, data curation, supervision, resources. **Francisca Joyce Elmiro Timbó de Andrade**: conceptualization, investigation, funding acquisition, writing – original draft, methodology, validation, visualization, writing – review and editing, software, formal analysis, project administration, data curation, supervision, resources.

## Conflicts of Interest

The authors declare no conflicts of interest.
